# An Adjuvanted Vaccine-Induced Pathogenesis Following Influenza Virus Infection

**DOI:** 10.3390/vaccines12060569

**Published:** 2024-05-23

**Authors:** Shiou-Chih Hsu, Kun-Hsien Lin, Yung-Chieh Tseng, Yang-Yu Cheng, Hsiu-Hua Ma, Ying-Chun Chen, Jia-Tsrong Jan, Chung-Yi Wu, Che Ma

**Affiliations:** 1 Genomics Research Center, Academia Sinica, 128 Academia Road, Section 2, Nankang, Taipei 115201, Taiwan; khlin@gate.sinica.edu.tw (K.-H.L.); jtseng@gate.sinica.edu.tw (Y.-C.T.); x32966@gmail.com (Y.-Y.C.); kib55916@gate.sinica.edu.tw (H.-H.M.); tsrong33@gate.sinica.edu.tw (J.-T.J.); cyiwu@gate.sinica.edu.tw (C.-Y.W.); cma@gate.sinica.edu.tw (C.M.); 2 Institute of Cellular and Organismic Biology, Academia Sinica, 128 Academia Road, Section 2, Nankang, Taipei 115201, Taiwan; onlychun@gmail.com

**Keywords:** adjuvant, vaccine, NKT cells, influenza virus, pathogenesis, precision medicine

## Abstract

An incomplete Freund’s adjuvant elicited an overt pathogenesis in vaccinated mice following the intranasal challenge of A/California/07/2009 (H1N1) virus despite the induction of a higher specific antibody titer than other adjuvanted formulations. Aluminum hydroxide adjuvants have not induced any pathogenic signs in a variety of formulations with glycolipids. A glycolipid, α-galactosyl ceramide, improved a stimulatory effect of distinct adjuvanted formulations on an anti-influenza A antibody response. In contrast to α-galactosyl ceramide, its synthetic analogue C34 was antagonistic toward a stimulatory effect of an aluminum hydroxide adjuvant on a specific antibody response. The aluminum hydroxide adjuvant alone could confer complete vaccine-induced protection against mortality as well as morbidity caused by a lethal challenge of the same strain of an influenza A virus. The research results indicated that adjuvants could reshape immune responses either to improve vaccine-induced immunity or to provoke an unexpected pathogenic consequence. On the basis of these observations, this research connotes the prominence to develop a precision adjuvant for innocuous vaccination aimed at generating a protective immunity without aberrant responses.

## 1. Introduction

The majority of vaccines require an exogenous adjuvant to induce an effective immune response against diverse pathogens despite an inferior response elicited by a peptide vaccine without an adjuvant [1]. It is a complex challenge to design a recombinant protein vaccine with built-in adjuvanticity, in particular for the consideration of antigen conformational stability [2,3,4,5]. In very few examples, a designed recombinant fusion vaccine has been able to engender substantial protection without an adjuvant [2]. The drawback and side effects are not clarified in those vaccines with built-in adjuvanticity, which are not applicable in all cases to protect against diverse diseases [2,4,6,7,8]. A nanoparticle vaccination represents the most obvious example to elicit an effective immune response without formulation in an adjuvant [9]. In fact, a variety of adjuvants have been incorporated into a nanoparticle vaccination to improve a protective response to varied viruses for humans, for instance using vaccines against ‘Hepatitis B virus’ (HBV), ‘Human papillomaviruses’ (HPV), and ‘SARS-CoV-2’ [10,11,12]. Many licensed adjuvants are currently available from pharmaceutical manufacturers [13,14,15]. The reason for development of an adjuvanted vaccine is that a larger quantity of antigens is generally required for generating a protective immunity without an exogenous adjuvant [1,15].

Seasonal influenza viruses have annually caused respiratory infectious diseases worldwide. Genetic re-assortment and drifted mutations are the mechanisms of a pandemic or an epidemic strain to escape from immune recognition [5,16,17]. The pandemic of influenza in 1918 was a notorious event that killed more people than those who died in the World War One [18]. Although not as devastating as the pandemic in 1918, there was a recent pandemic outbreak in 2009, which still resulted in a meaningful mortality of infected entities [17]. There is the requirement of an effective strategy for the emergent production of sufficient doses of vaccines to confront the next pandemic outbreak. A better adjuvant formulation is a good option to induce a protective response with a reduced dosage of antigens, which could potentially alleviate the pressure of a global need for vaccination to cope with a pandemic outbreak [13,15,19].

For decades, water in oil (w/o) emulsions like Freund’s adjuvants have been widely exploited to enhance the immunogenicity of all kinds of antigens. Freund’s adjuvants are doubtless considered to be a good option for research to elicit a better immune response, even if alternatives have been commercially available for a while. The problems of those commercial products are that they are neither practical enough for antigen incorporation nor steady enough as Freund’s adjuvants to produce a reproducible result in an immunization experiment [20,21]. The incomplete Freund’s adjuvant (IFA) has been employed in clinical trials of immunotherapy in humans. It has been very successful to induce a potent CD8 T cell activity to improve the survival rate of patients in a very late stage of melanoma or in the metastatic stage of non-small-cell lung cancer [22]. Freund’s adjuvants could not be implemented in research for human subjects due to their pathogenic effects, particularly those of the complete Freund’s adjuvant (CFA). Glycosylation could influence antigen immunogenicity, which indicates a significant effect of glycan on immune responses [23,24]. It is critical to estimate the biological function of varied glycan-formulated adjuvants. Natural killer T (NKT) cells are activated following the recognition of glycolipids presented by non-classical MHC molecules, such as CD1 [25]. After stimulation with α-galactosyl ceramide (α-GC), NKT cells secrete cytokines to potentiate antigen-presenting cells (APCs) via cytokine circuits [26]. Resembling α-GC, its glycolipid derivatives would be inherently able to condition APCs to reshape a vaccine-induced immunity.

As a critical constituent of vaccination materials, the pathogenesis of adjuvants has not been extensively researched. Despite several vaccination materials licensed for human use, continued reports of adverse effects from all types of vaccines have revealed safety concerns on those licensed materials. Even with delivery systems retaining intrinsic adjuvanticity, it would be unexceptional for them to lack a privileged status [6,27]. A trial-and-error way used to be exploited in the development of new vaccination materials. The scientific approach should be meditated to be employed in these research fields. We aimed to evaluate the hypothesis that adjuvant formulations with various glycolipids could have a significant impact on vaccine-induced responses. The first assumption was that certain glycolipids would enhance vaccine-induced immunity. The second was for certain glycolipids to provoke aberrant responses following vaccination. At the same time, we could develop new types of adjuvant formulations compatible for use in humans. The optimized formulation of a prevalent adjuvant, like an aluminum hydroxide adjuvant (AH), was reinvigorated for innocuous vaccination as a harmless alternative of Freund’s adjuvants [6,13,14,27,28].

## 2. Materials and Methods

### 2.1. The Sources of Glycolipids, α-GC, and C34

The α-GC was purchased from Chemical/Biochemical manufacturers (Sigma-Aldrich Co., St. Louis, MO, USA). Several intermediate analogues with aromatic modifications at an acyl chain of α-GC were synthesized by use of an organic chemistry approach to investigate their capability to transform immune cells, which were unofficially and incorrectly designated as phenyl α-GC. They were actually aromatic derivatives of α-GC. One of them, designated as C34, was synthesized on a laboratory-scale for sensible utilization in immunization experiments. The glycolipids were analyzed with a Zetasizer Nano ZS (Malvern instruments Ltd., Malvern, UK) and mass spectrometry to confirm their particulate size as well as to meet a standard requirement of their physiochemical properties and structures.

### 2.2. The Influenza Virus and an Inactivated Vaccine

The influenza virus, A/California/07/2009 (H1N1), was first isolated by the CDC, USA in 2009. The New York Medical College, USA, has developed a vaccine strain NYMC X-181, derived from an influenza A/California/07/2009 virus (H1N1). An influenza A virus was propagated and quantified in Madin–Darby Canine Kidney (MDCK) cells (A vaccine strain NYMC-181) and MDCK cells obtained from Genomics Research Center (GRC)/Academia Sinica/TAIWAN, Adimmune Co. (Taichung City, Taiwan)/TAIWAN and National Defense Medical College/TAIWAN as stated by colleagues, originated from ATCC, USA). The 50% tissue culture infectious dose (TCID_50_) was determined by a serial dilution of the virus stock in the MDCK cells.

A virus stock of the NYMC X-181 vaccine strain was propagated in a specific pathogen-free (SPF) chicken embryo of 9–10 days old. An infectious allantoic fluid was collected from infected SPF eggs to be further purified and enriched with sucrose-gradient ultracentrifugation. To inactivate a propagated virus stock as the source of vaccine preparation, a purified vaccine strain was incubated with a formalin to eliminate its infectivity as well as replication capacity. For quantification, numerous assays were employed to titrate a purified virus stock as well as to quantify the inactivated vaccine strain by a hemagglutination unit (HAU) or by the quantity of hemagglutinin (HA) (mg/mL). The size of the inactivated virion vaccine was measured and confirmed with a Zetasizer Nano ZS (Malvern instruments Ltd.) at GRC, Academia Sinica, Taiwan, R.O.C.

### 2.3. The Experimental Animals

BALB/c mice were obtained from a pathogen-free facility located at the National Laboratory Animal Center or from Biolasco Taiwan Co., Ltd., Nankang, Taipei city, Taiwan. Any experiment in the animal facility was performed by a well-trained veterinarian at the Institute of Cellular and Organismic Biology (ICOB), Academia Sinica, Taiwan, R.O.C. A protection experiment was completed at biosafety-level-2 and -3 laboratories, GRC, Academia Sinica, Taiwan, R.O.C.

### 2.4. Neutralization Assay

An amount of 4–5 mice of each experimental group was immunized to evaluate the neutralizing activity of every serum sample derived from individual immunized mice. The neutralizing activity of serum from immunized mice was analyzed by a micro-neutralization assay with the A/California/07/2009 (H1N1) virus in MDCK cells. We titrated serum samples to be added into each well with 100 TCID_50_ of an A/California/07/2009 (H1N1) virus. Following an interaction between diluted serum samples and the influenza A virus at 37 °C in the incubator, a mixture of diluted serum and virus was transferred onto MDCK cells in a 96-welled plate in a CO2 incubator for the daily observation of a cytopathic effect (CPE) in each well. The neutralizing activity was referred to the dilution factor of serum samples, based on the viral titer to achieve the TCID_50_.

### 2.5. Immuno-Microarray Assay

An amount of 4–5 mice of each experimental group was immunized to assess the level of specific antibody titer of every serum sample derived from individual immunized mice. A recombinant HA and an inactivated influenza antigen were dissolved and titrated in sodium phosphate buffer or phosphate buffer saline. A serial dilution of a recombinant HA or an inactivated influenza antigen was spotted on a microarray slide to detect the level of the influenza A virus, H1N1-specific antibody response (AD3200, BioDot Inc., Irvine, CA, USA). Following the interaction of a microarray slide in diluted serum samples, the dyelight 650-labeled anti-IgG antibody bound to the influenza A virus, H1N1-specific antibody to reveal fluorescent signals on a spotted slide. A microarray slide was then scanned by a laser scanner to count the fluorescent intensity of the reaction sample (GenePix 4300A, Molecular Devices, San Jose, CA, USA).

All data were presented as an average of ten independent spotting experiments. The total fluorescence intensity was presented as mean ± standard deviation after normalization with a background signal from a control group. Ten independently reproduced experiments were employed on each dilution of a spotted antigen to be statistically significant for comparison between groups. The statistical program was exploited for data analysis.

### 2.6. Protection Experiments

An amount of 5 BALB/c mice of each experimental group was immunized subcutaneously (s.c.) 2–3 times with an inactivated influenza vaccine formulated in a variety of adjuvants at 3-week intervals. Immunized mice were then inoculated via an intranasal route with 10-times 50% mouse lethal dose (MLD_50_) of an A/California/07/2009 (H1N1) virus per mouse after the final immunization. Following the lethal challenge with the same strain of the influenza A virus, the mortality and morbidity of the mice were recorded daily to evaluate the efficacy of each adjuvant. The survival rate, percentage of weight loss, and pathogenic indices were recorded daily until 14–16 days subsequent to the lethal challenge of the same virus. A similar experimental procedure was repeated in another independent experiment. According to the criteria established by the Institutional Animal Care and Use Committee (IACUC), infected mice were terminated with humane euthanasia if the weight loss was beyond 20% with concern to other pain indices at the same time.

## 3. Results

### 3.1. The Influenza-A-Virus-Specific Antibody Response Induced by Distinct Vaccine Formulations in Freund’s Adjuvants

**Figure 1:**
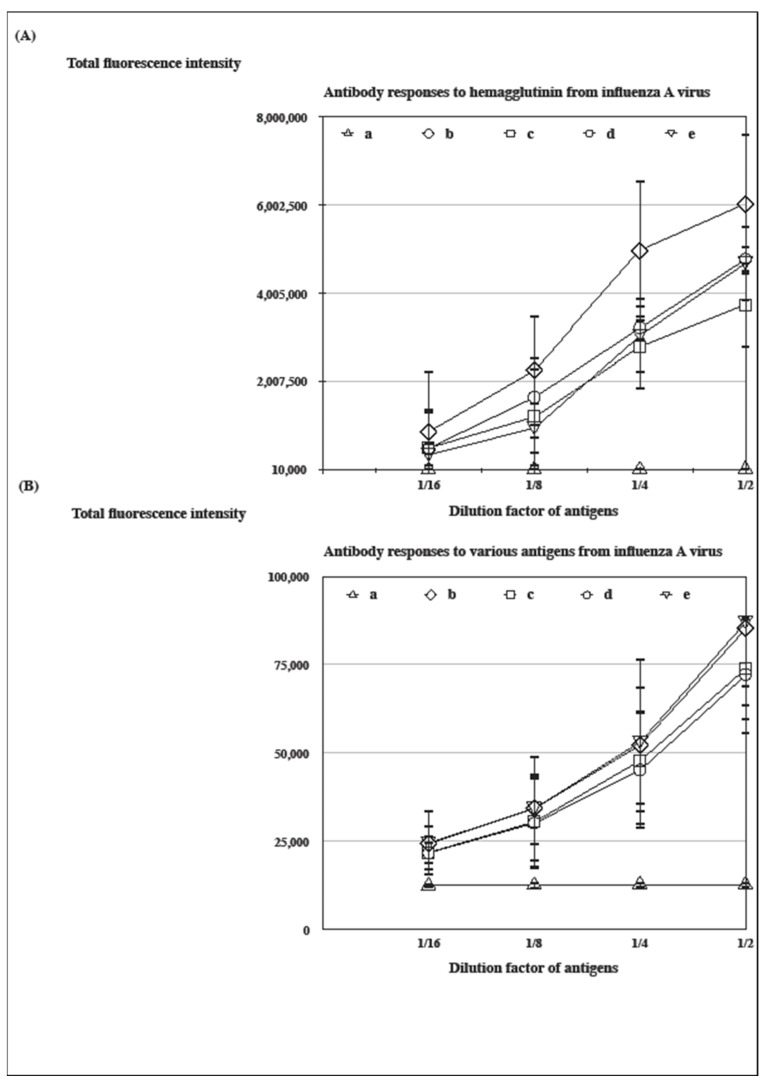
The influenza-A-virus-specific antibody response induced by distinct vaccine formulations in Freund’s adjuvants. BALB/c mice were immunized s.c. with △: IFA as a control group (a), ◇: CFA plus an antigen (b), □: IFA plus an antigen (c), ○: IFA plus an antigen formulated with α-GC (d), and ▽: IFA plus an antigen formulated with C34 (e). Sera were collected from immunized mice to detect a specific antibody response with a microarray assay by a serial dilution of (**A**) a recombinant HA or (**B**) an inactivated influenza antigen. The results were reproducible at least in two independent experiments (Student’s *T* test, *p* < 0.005 at 1:2 dilution of a recombinant HA).

BALB/c mice were immunized with an inactivated vaccine strain of NYMC-X181 emulsified in an IFA formulated with distinct glycolipids (w/o emulsions, Figure 1A) [29]. The recombinant HA was derived from an A/California/07/2009 (H1N1) virus to detect an influenza-A-virus-specific antibody response with a microarray assay. The glycolipids, α-GC and its modified biochemical C34, were formulated with an IFA to evaluate their potential to improve the immunogenicity of an adjuvanted inactivated vaccine (c, d, and e in Figure 1A). The CFA-formulated vaccine induced a superior antibody response than any of the other immunized groups (*p* < 0.0001 between groups b and c at 1:2 dilution of a recombinant HA in Figure 1A). Glycolipids improved the immunogenicity of the adjuvanted inactivated vaccine following immunization (*p* < 0.005 between groups d and c, *p* < 0.0001 between groups e and c at 1:2 dilution of a recombinant HA in Figure 1A). The results were reproducible at least in two independent experiments.

The sensitivity to detect the level of a specific antibody response was significantly affected by the types of antigens used in the experiments. Inactivated antigens represented an inferior fluorescence signal than a recombinant HA even if both of them gave similar experimental results (*p* < 0.0001 between groups b and c at 1:2 dilution of an inactivated antigen in Figure 1B). The data were analyzed with Student’s *T* test.

### 3.2. The Influenza-A-Virus-Specific Neutralizing Antibody Activity Induced by Distinct Vaccine Formulations in Freund’s Adjuvants

**Figure 2:**
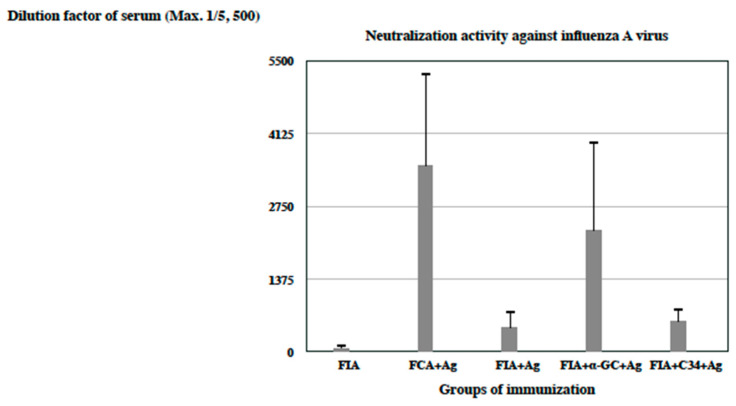
The neutralizing antibody activity induced by distinct vaccine formulations in Freund’s adjuvants. BALB/c mice were immunized s.c. with CFA plus an antigen (CFA + Ag), IFA plus an antigen (IFA + Ag), IFA plus an antigen formulated with α-GC (IFA + α-GC + Ag), IFA plus an antigen formulated with C34 (IFA + C34 + Ag), and IFA as a control group (IFA). Sera were collected from immunized mice to analyze the neutralizing activity against an influenza A virus in MDCK cells (ANOVA analysis, *p* < 0.001).

Sera from immunized BALB/c mice were tested to semi-quantify an in vitro neutralizing activity (NA) against an A/California/07/2009 (H1N1) virus (Figure 2). The CFA-formulated vaccine elicited a superior neutralizing activity compared with the IFA-formulated vaccine (CFA + Ag versus IFA + Ag). The glycolipid α-GC was able to improve the neutralizing activity induced by the IFA-formulated vaccine, which was analogous to that generated by a CFA-formulated one (CFA + Ag versus IFA + α-GC + Ag). The modified glycolipid C34 was not equivalent to α-GC for the induction of a comparable neutralizing activity following emulsion in an IFA-formulated vaccine (IFA + α-GC + Ag versus IFA + C34 + Ag). The result of multiple comparisons between groups was statistically significant with an ANOVA analysis (*p* < 0.001).

### 3.3. Protection against Mortality Induced by Distinct Vaccine Formulations in Freund’s Adjuvants Following an Intranasal Challenge of A/California/07/2009 (H1N1) Virus

**Figure 3:**
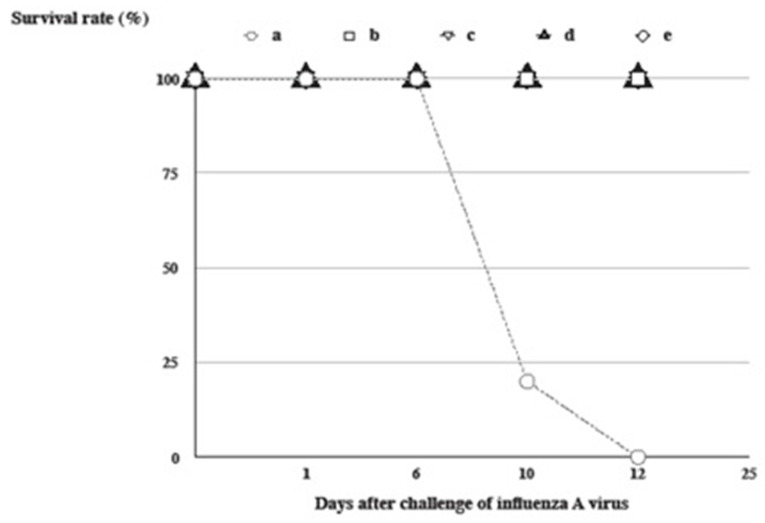
Protection against the influenza A virus induced by distinct vaccine formulations in Freund’s adjuvants. BALB/c mice were immunized s.c. with ○: IFA as a control group (a), □: CFA plus an antigen (b), ▽: IFA plus an antigen (c), ▲: IFA plus an antigen formulated with α-GC (d), and ◇: IFA plus an antigen formulated with C34 (e). Following the final immunization, immunized mice were challenged with A/California/07/2009 (H1N1 virus). Mortality was recorded daily subsequent to a lethal challenge of influenza A virus.

BALB/c mice were infected with 10-times MLD_50_ of the A/California/07/2009 (H1N1) virus via an intranasal route after immunization. The mortality was recorded daily until 14 days after a lethal challenge of influenza A virus (Figure 3). Immunized groups of mice were protected against mortality at a survival rate of 100% in comparison with the IFA-immunized control group (a, b, c, d, and e in Figure 3). The rate of weight loss was not statistically significant between vaccine-immunized groups except for the IFA-immunized control group.

### 3.4. The Influenza-A-Virus-HA-Specific Antibody Response Induced by Distinct Vaccine Formulations in Aluminum Hydroxide Adjuvant

**Figure 4:**
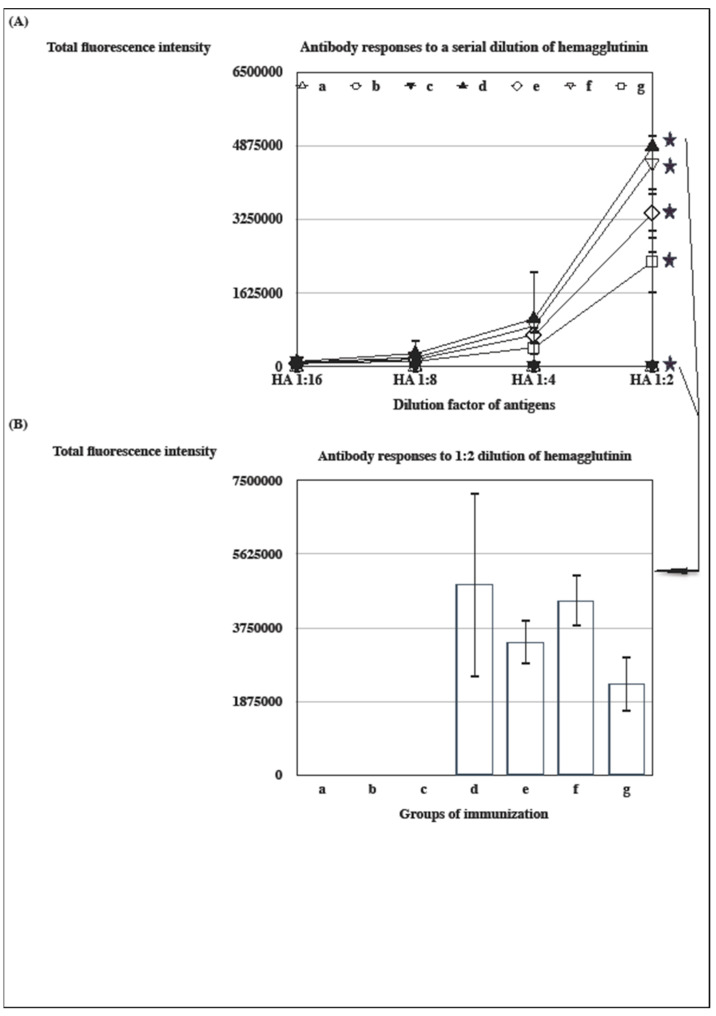
The antibody response to a recombinant influenza hemagglutinin induced by distinct vaccine formulations in an aluminum hydroxide adjuvant. BALB/c mice were immunized s.c. with △: PBS as a control group (a), ○: IFA (b), ▼: AH (c), ▲: IFA plus antigens (d), ◇: AH plus antigens (e), ▽: AH plus antigens formulated with α-GC (f), and □: AH plus antigens formulated with C34 (g). Sera were collected from immunized mice to detect specific antibodies with a microarray assay. Antibody responses to a serial dilution of a recombinant HA (**A**) and to the same antigen at 1:2 dilution (**B**) (ANOVA analysis, *p* < 0.05 at 1:2 dilution of a recombinant HA). 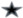 The result of antibody responses to 1:2 dilution of a recombinant HA.

The AH was reformulated with glycolipids, α-GC or C34, to improve the immunogenicity of an adjuvanted inactivated vaccine (Figure 4). Influenza-A-virus-HA-specific antibody responses were measured with a microarray assay after the immunization of an inactivated vaccine in distinct adjuvanted formulations. The α-GC improved the immunogenicity of an inactivated vaccine following formulation in an aluminum hydroxide adjuvant (c, e, and f in Figure 4A,B). The α-GC-formulated AH (AH-α-GC) was efficacious in the induction of an equivalent antibody response as an IFA (d, e, and f in Figure 4A,B). By contrast, the C34-formulated AH (AH-C34) antagonized the stimulatory effect of the AH on a specific antibody response that was generated with the same source as the inactivated vaccine (e, f, and g in Figure 4A,B). The result of the ANOVA analysis was significant for multiple comparisons of all groups (*p* < 0.05).

### 3.5. Protection against Mortality and Morbidity Induced by Distinct Vaccine Formulations in an Aluminum Hydroxide Adjuvant Following an Intranasal Challenge of A/California/07/2009 (H1N1) Virus

**Figure 5:**
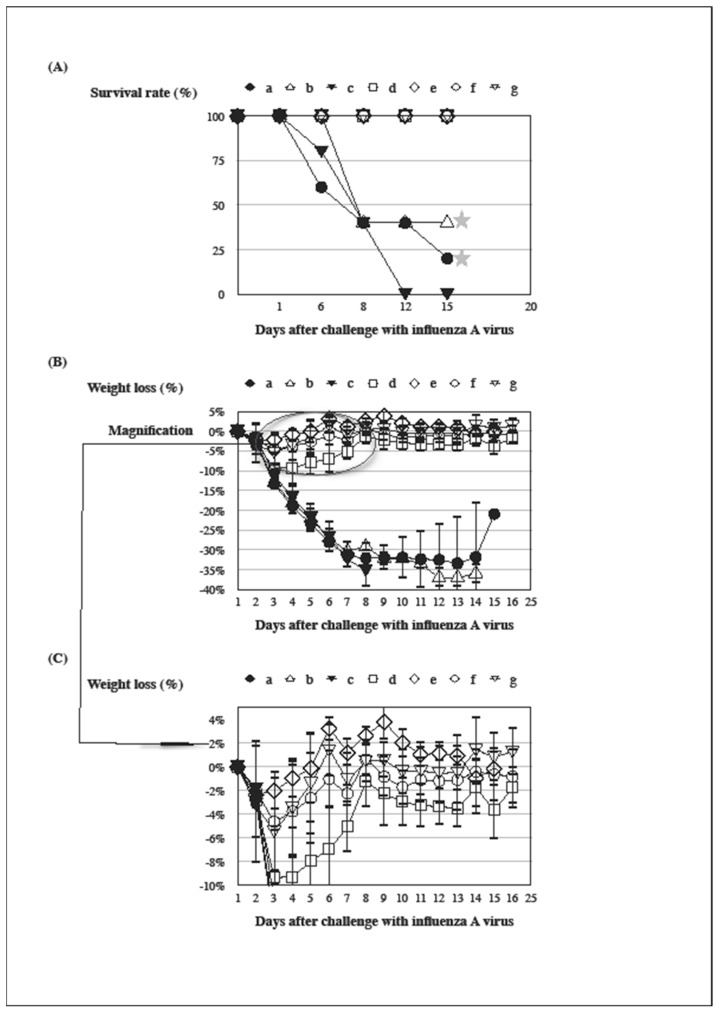
Protection against mortality and morbidity induced by distinct vaccine formulations in an aluminum hydroxide adjuvant following infection with the A/California/07/2009 (H1N1) virus. BALB/c mice were immunized s.c. with ●: PBS as a control group (a), △: IFA (b), ▼: AH (c), □: IFA plus antigens (d), ◇: AH plus antigens (e), ○: AH plus antigens formulated with α-GC (f), and ▽: AH plus antigens formulated with C34 (g). Immunized mice were challenged with an A/California/07/2009 (H1N1) virus following the final immunization at a 3-week interval. Mortality (**A**) and morbidity (**B**,**C**) were observed and recorded daily after a lethal challenge of the A/California/07/2009 virus. (**C**) was the magnified version of (**B**) from day 2 to 9. A similar result was reproducible in another independent experiment (ANOVA analysis from day 4 to 9, *p* < 0.05). 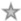 Three virus-infected mice in group a and b had weight loss beyond 20% on day 15. They were terminated with humane euthanasia on day 16 with concern to other pain indices. □: The ruffled fur and slower locomotion were observable pathogenic signs in the group immunized with an IFA plus antigens (d).

BALB/c mice were immunized with a variety of adjuvanted inactivated vaccines in AH to evaluate their protective efficacy, for instance IFA, AH, AH-α-GC, and AH-C34 (d, e, f, and g in Figure 5A). All vaccine-immunized groups protected against a lethal challenge of the A/California/07/2009 (H1N1) virus in comparison with control groups (a, b, c, d, e, f, and g in Figure 5A). The group immunized with an IFA-formulated vaccine resulted in a significant weight loss from day 2 to 9 following a lethal challenge of the same virus (d in Figure 5B,C). An inactivated vaccine formulated in varied AHs did not provoke an obvious weight loss of immunized groups from day 3 to 9 (e, f, and g in Figure 5B,C), which was induced by an intranasal challenge of the same virus (d in Figure 5B,C). The ruffled fur and slower locomotion were observable pathogenic signs in the group immunized with an IFA-formulated vaccine (d in Figure 5C, refer to criteria established by IACUC). Those groups immunized with varied AH-formulated vaccines presented none of pathogenic signs as observed in the IFA-antigen-immunized one. Similar results were reproducible in another independent experiment. The result of the ANOVA analysis was significant for all groups from day 4 to 9 (*p* < 0.05).

## 4. Discussion

It has exceeded 43 years since a smallpox epidemic was completely eliminated via a vaccination campaign of the World Health Organization, which was attributed to a live attenuated vaccine originally introduced by Dr. Edward Jenner. Nevertheless, it was inevitable that several vaccine-associated incidents were elicited by live attenuated vaccines from the 20th to 21st centuries [30,31]. The disadvantage of a live attenuated virus strain is the risk of reverting into a wild-type-like pathogenic one. A recent outbreak of circulating vaccine-derived poliovirus (cVDPV) represented an obviously dangerous signal to an issue of public health for the whole world [31]. In spite of the deprivation of their evolving capacity, various types of inactivated vaccines have also provoked a vaccine-associated pathogenesis in vaccinees [32,33,34]. The quality of vaccine-induced responses and autoimmunity could not be left out as etiological factors of these vaccine-related incidents.

Since 2009, a global vaccination program to halt the pandemic outbreak of swine-origin influenza A (H1N1) virus has represented a disenchanting circumstance for an adjuvanted vaccination. The illness of narcolepsy was linked to the part of a global vaccination program in certain countries, peculiarly in Sweden [33]. An adjuvant was suggested to be one of those factors to mediate this special and rare illness. Later, the autoimmunity of a nervous system was uncovered to be the major reason responsible for this vaccine-induced disorder. It was very likely to be induced by an antigen itself despite the absence of a convincing and conclusive research result (doi: 10.1126/scitranslmed.3009995). The enhancement of an immune response to an autoantigen could not be omitted in those events following re-vaccination of infected entities with an adjuvanted vaccine [13,19,33]. Freund’s adjuvants are the most well-known and effective oil-based adjuvants for use in successful trials against either malignancies or viruses [22,35]. However potent the CFA is, a side effect will bring out a severe granulomatous reaction in tissues and organs in immunized animals [36]. A few oil-based and lipophilic adjuvants were actually implicated in numerous incidents of vaccine-associated pathogeneses in clinical trials [33,37,38]. Regarding a global supply chain to mitigate a pandemic outbreak, antigen sparing for vaccination was considered to outweigh the risk of pathogenesis caused by an adjuvanted vaccine [15]. In fact, a potent adjuvant ought to be universally used in a variety of antigens without causing a pathological outcome. As a prospective object, it is no longer a redundant task for both immunologists and vaccinologists to develop innocuous adjuvants for use in humans.

NKT cells express invariant T cell receptors (TCRs) to regulate immune responses by cytokine networks. Glycolipids, such as α-GC, are able to activate NKT cells via presentation by CD1d on APCs. NKT cells have been proposed to be the interplay between innate and adaptive immune systems, extensively for B-cell responses [39]. These special characteristics have prompted us to develop new adjuvanted formulations with glycolipids. AH has been selected for this research in addition to Freund’s adjuvants given that it has been licensed for use in humans for years due to its magnificent safety records [40]. We have exploited two types of glycolipids, α-GC and its synthetic analogue C34 [41], to see if they have a pronounced impact on an immune system after formulation in IFA or AH.

From the results of Figure 1, Figure 2, Figure 3, Figure 4 and Figure 5, glycolipids potentiated the adjuvant effect on a specific antibody response following formulation in IFA or AH, except AH-C34, which was semi-quantitated with immuno-microarrays [42]. Both IFA and AH formulated with α-GC continued to raise a higher HA-specific antibody response as well as a more effective neutralizing activity than those without glycolipid. On the other hand, C34 was not as efficacious in the induction of an equivalent antibody response as was α-GC but antagonized the adjuvant effect of AH. The α-GC activated NKT cells to secrete both T helper 1 (Th1) and T helper 2 (Th2) cytokines, while C34 was suggested to be a Th1-activator [24]. It is likely that vaccine-induced immunity was skewed to more Th1-oriented responses by C34 [43]. By contrast, α-GC was oriented in both ways without compromising its potential to elevate the efficacy of adjuvanted vaccination [26]. Since certain trial results have disapproved of the paradigm role of Th1/Th2 model, the regulatory properties of invariant TCRs in NKT cells could provide a supplemental interpretation for these experimental observations [26,39]. It was not anticipated that groups of mice revealed a complete protection against mortality with a decreased level of antibody titer and an inferior neutralizing activity, like the AH-C34-antigen (titer), IFA-antigen (NA), and IFA-C34-antigen (NA) (Figure 2, Figure 3, Figure 4 and Figure 5). Antibody titer and neutralizing activities may not be the main protective mediators in these protected groups. Other biological mechanisms, like the virulence of a vaccine strain, an antibody affinity, and T cell activities, should all be considered to be complementary factors mediating a complete protection in vivo [29,44,45,46,47].

Furthermore, it was quite unexpected that the group of mice immunized with an IFA-antigen brought out a more profound weight loss than other immunized ones in the presence of higher antibody titer (d in Figure 5C) (graphic abstract). Groups of mice immunized with aluminum-containing formulations recovered much earlier from weight loss than did the IFA-antigen-immunized group (d, e, f, g, in Figure 5C). To our surprise, glycolipids formulated in the aluminum hydroxide adjuvant did not provoke an obvious weight loss in those immunized groups. Vaccine-associated enhanced respiratory disease (VARED) has been reported in several infectious diseases, which has motivated scientists to develop new immunization ingredients and strategies [32,48]. VARED occurred when vaccinated animals were challenged with a heterologous influenza virus in the presence of non-neutralizing antibodies [47,48]. The AH-GC-antigen group elicited an equivalent antibody titer as the IFA-antigen-immunized one (d and f in Figure 4B). By contrast, the AH-C34-antigen induced a lower level of antibody titer than an IFA-antigen (d and g in Figure 4B). Thus, the antibody titer was unlikely to be a factor to antagonize morbidity observed in the IFA-antigen-immunized group. Other mechanisms were possibly responsible for weight loss and other pathogenic signs following the intranasal challenge of a live virus. Antibody-dependent enhancement (ADE) has been suggested to mediate the augmentation of newly serotyped infections of heterotypic dengue viruses, which could contribute to the increased risk of aggravated disease [44,45]. Moreover, immune complexes were proposed to provoke a vaccine-enhanced illness in respiratory syncytial virus (RSV) infection. The vaccine-enhanced illness was attributed to non-neutralizing and low-affinity antibody responses [44,47]. In comparison with the other groups in this research, the IFA-antigen-immunized group not only elicited a comparable level of antibody titer to bind to an influenza HA antigen (b, d, f in Figure 4B), but raised minor activity in neutralizing a live influenza virus (Figure 2). Antibody affinity is likely to be the sole mediator for this pathogenic event [3,5,7]. Alternatively, the inactivated vaccine was regarded as notorious for activating a negligible CD8 T cell activity, especially for those formulated in certain adjuvants [32,46]. The absence of CD8 T cell activity could be another factor in inducing an unexpected pathogenic outcome. From these research results, we unveiled that the improper use of adjuvants could be one of the major reasons causing vaccine-induced morbidity (Figure 5C). These results demonstrated that exogenous adjuvants and built-in adjuvanticity do not simply augment a vaccine-induced immunity but also potentially irritate an unpredictable pathogenesis to circumvent foreknowledge tools from subjects regarding computational modeling [2,6,16,27,28,32,33,34,37,38,47]. The correlated computing programs failed to predict this unanticipated outcome involving the use of adjuvants, for instance programs equipped with ‘deep learning’ or with the style of ‘artificial general intelligence (AGI)’ [3,4,5,16].

From the vantage point of immunological significance, a glycolipid could be the optimal component, like α-GC, in order to upregulate antibody titer (Figure 1 and Figure 4). But we could be misled by a rational design of vaccination to prompt a superior antibody titer by sparing other correlates of pathogenesis, for instance the quality of immune responses (Figure 2, Figure 4 and Figure 5) [45,46,47]. Based on results from Figure 2, Figure 4 and Figure 5C, IFA-antigen raised a significant level of specific antibody titer but a minor level of neutralizing activity against the influenza virus (graphic abstract). An inactivated vaccine actually contains a variety of viral antigens to induce many types of immune responses. In this research, it would be inappropriate to make a conclusive statement that pathogenic mechanisms are simply mediated by one type of adjuvanted vaccine-induced immune response. There will be no limitation for prospects of future research projects to further elucidate detailed pathogenic mechanisms induced by vaccination.

For many pathogenic viruses without available vaccines, a balanced immunity consisting of both humoral and cellular responses is an indispensable requirement for a complete protection [26]. C34 should be discounted for its particular property to suppress a specific antibody response by design of modification. As suggested, C34 and other related analogues are more likely to be candidates to generate cell-mediated immunity against intracellular mycobacteria. Nonetheless, glycolipid-induced hepatitis was an unwanted defect in a clinical trial of human subjects [37], which should be taken into consideration for adjuvant development. The current paradigm in ‘Immunology’ suggests the existence of polarized CD4^+^ human T cells (Th) based on their profile of cytokine secretion. Th1 cells produce interferon-gamma, interleukin-2, and tumor necrosis factor-beta, which mediate cellular immunity. By contrast, Th2 cells produce IL-4, IL-5, IL-10, and IL-13, responsible for strong antibody production and eosinophil activation. Th1 cells mainly develop following infections by intracellular bacteria and viruses, whereas Th2 cells predominate in response to infestations by gastrointestinal nematodes [43]. Recently, remarkable achievements in clinical trials for the utilization of adjuvanted vaccinations have turned this paradigm into a contested status, re-emphasizing the capacity of adjuvants to skew vaccine-induced immunity toward Th2 responses against obligately intracellular pathogens, such as viruses [11,14,43]. Thus, the above-mentioned results of glycolipid immunization may not be well-interpreted with the paradigm of a Th1/Th2 protection model. The detailed mechanism with respect to a higher antibody titer induced by an AH-GC-antigen could be more complex than Th2-oriented immune responses exemplified by the Th1/Th2 protection model (Figure 2 and Figure 4). The NKT cell is actually able to exert innate signals directly on B lymphocytes [39]. In this research, there was not a noticeable weight loss or any pathogenic signs in adjuvanted glycolipid-immunized mice (Figure 5C). Glycolipid thus bestows a benefit on us as a formulation ingredient to develop a contemporary substitute of Freund’s adjuvants. Glycolipids could be dispensable for adjuvanted formulations, considering protection versus pathogenesis, given that the AH-antigen-immunized group achieved the same level of risk-free protection without glycolipid (Figure 5). Testing the hypothesis with adjuvanted glycolipid vaccinations has brought us back to a scientific route to design a safer, more precise, and group-oriented vaccination instead of a simply rational way, in particular with respect to pathogenesis [6,27,30,31,32,33,34].

Following the successful trials to protect the elderly, two similar vaccines developed by advanced recombinant technologies have been taken to further steps to be exploited for ‘maternal immunization’ against RSV infections in infants. A trial using a prefusion protein vaccine (PreF3, Arexvy) was immediately halted over a rise in preterm births and neonate deaths [28]. Another trial with a similar bivalent prefusion protein vaccine (RSVpreF, Abrysvo) was claimed to be effective against medically attended severe RSV-associated lower respiratory tract illness in infants without significant pathogenic outcomes [49]. Until now, the etiological factors concerning this aberrant consequence have been interpreted in too vague a way to discern the discrepant results between the two vaccine trials [14,28,49]. The main difference between the two vaccines consists in their vaccination constituents. The one halted over a rise in preterm births was an adjuvanted vaccination trial against RSV infections in infants, while the other trial contained no adjuvant. It would be improper to conclude that an adjuvant was really the cause for this vaccine-related incident, based on insufficient, insubstantial, and unconvincing scientific proofs [28]. Again, these successful trial results to protect the elderly disprove the paradigm of the Th1/Th2 protection model, since the adjuvant employed in trials has the potential to trigger Th2-oriented responses in some vaccinees [9,10,11,12,50]. As a critical component of vaccination materials, the adjuvant should not be neglected as a possible etiological factor in this event ([32,51], Figure 5C).

## 5. Conclusions

This research indicated that adjuvants are not only apt to improve vaccine-induced immunity but likely to result in an abrupt pathogenesis. On the basis of this observation, we will ultimately have a broader scientific way to design not simply an antigen itself but an adjuvant and other vaccine-delivery systems. The precision vaccination approach would surmount the potential risk of imperfect vaccines causing an unpredictable pathogenesis in the future [8,52,53,54,55,56,57].

## Data Availability

The authors declare that data will be made available from ‘GRC, Academia sinica, Taiwan, R.O.C.’ upon request.
